# Supplementary material for: Classifying T cell activity in autofluorescence intensity images with convolutional neural networks
Source: J Biophotonics. 2019 Dec 15;13(3):e201960050. doi: 10.1002/jbio.201960050 (PMC7065628; doi:10.1002/jbio.201960050)
Supplement: Supplementary file 1 — Appendix S1. Supporting information. [file JBIO-13-e201960050-s001.pdf]

Classifying T cell activity in autofluorescence intensity images with convolutional neural networks: supplementary information

Zijie J. Wang[1,2,*], Alex J. Walsh[2,+], Melissa C. Skala[2,3], and Anthony Gitter[1,2,4]

[1]Department of Computer Sciences, University of Wisconsin-Madison, Madison, Wisconsin
[2]Morgridge Institute for Research, Madison, Wisconsin
[3]Department of Biomedical Engineering, University of Wisconsin-Madison, Madison, Wisconsin
[4]Department of Biostatistics and Medical Informatics, University of Wisconsin-Madison, Madison, Wisconsin
[*]Current address: School of Computational Science and Engineering, Georgia Institute of Technology, Atlanta, Georgia
[+]Current address: Department of Biomedical Engineering, Texas A&M University, College Station, Texas

Supplementary Tables and Figures

Donor	Accuracy	Precision	Recall	Average Precision	AUC	Activated Count	Quiescent Count
1	13.16%	13.16%	100.00%	13.16%	50.00%	235	1551
2	17.89%	0.00%	0.00%	82.11%	50.00%	647	141
3	73.52%	0.00%	0.00%	26.48%	50.00%	446	1238
5	26.48%	0.00%	0.00%	73.52%	50.00%	683	246
6	56.75%	0.00%	0.00%	43.25%	50.00%	442	580

Table S1. Performance of Frequency Classifier

Donor	Accuracy	Precision	Recall	Average Precision	AUC	Activated Count	Quiescent Count
1	84.60%	43.79%	60.00%	53.90%	82.27%	235	1551
2	68.02%	94.99%	64.45%	95.38%	81.61%	647	141
3	79.57%	61.75%	60.09%	68.67%	81.10%	446	1238
5	80.81%	88.12%	85.42%	95.20%	87.16%	683	246
6	80.68%	73.43%	86.97%	90.19%	90.59%	442	580

Table S2. Performance of Logistic Regression (Image Pixel Matrix)

Donor	Accuracy	Precision	Recall	Average Precision	AUC	Activated Count	Quiescent Count
1	88.13%	55.35%	50.64%	53.67%	79.92%	235	1551
2	65.99%	96.56%	60.74%	94.46%	79.34%	647	141
3	81.59%	68.89%	55.61%	68.12%	74.68%	446	1238
5	80.92%	89.32%	84.11%	95.20%	86.68%	683	246
6	83.02%	78.17%	84.49%	89.86%	89.02%	442	580

Table S3. Performance of Logistic Regression (Total Intensity and Mask Size)

Donor	Accuracy	Precision	Recall	Average Precision	AUC	Activated Count	Quiescent Count
1	95.74%	86.98%	79.57%	88.85%	95.61%	235	1551
2	76.65%	91.56%	78.83%	95.24%	82.33%	647	141
3	92.16%	94.60%	74.66%	93.07%	96.26%	446	1238
5	81.81%	81.81%	96.78%	93.74%	86.70%	683	246
6	89.33%	82.33%	95.93%	95.97%	97.01%	442	580

Table S4. Performance of Logistic Regression (CellProfiler Features)

Donor	Accuracy	Precision	Recall	Average Precision	AUC	Activated Count	Quiescent Count
1	88.80%	55.28%	75.74%	65.40%	90.55%	235	1551
2	82.87%	96.05%	82.69%	96.60%	88.78%	647	141
3	88.54%	82.14%	72.20%	84.59%	90.34%	446	1238
5	84.78%	85.58%	95.19%	96.48%	92.05%	683	246
6	87.41%	80.62%	93.48%	94.44%	95.36%	442	580

Table S5. Performance of One-layer Fully Connected Neural Network

Donor	Accuracy	Precision	Recall	Average Precision	AUC	Activated Count	Quiescent Count
1	94.23%	78.21%	77.87%	82.15%	95.36%	235	1551
2	87.06%	96.58%	87.33%	97.86%	91.92%	647	141
3	91.45%	95.76%	70.85%	91.55%	94.12%	446	1238
5	87.41%	87.83%	96.19%	96.40%	92.41%	683	246
6	87.38%	78.61%	97.29%	96.70%	97.36%	442	580

Table S6. Performance of LeNet CNN

Donor	Accuracy	Precision	Recall	Average Precision	AUC	Activated Count	Quiescent Count
1	94.57%	81.08%	76.60%	86.42%	95.96%	235	1551
2	90.10%	96.87%	90.88%	99.06%	95.89%	647	141
3	93.94%	93.22%	83.18%	94.08%	96.66%	446	1238
5	87.08%	87.09%	96.78%	96.83%	92.81%	683	246
6	86.11%	75.95%	99.32%	97.61%	98.49%	442	580

Table S7. Performance of Pre-trained CNN Off-the-shelf Model

Donor	Accuracy	Precision	Recall	Average Precision	AUC	Activated Count	Quiescent Count
1	96.81%	92.79%	82.13%	91.71%	95.70%	235	1551
2	91.88%	97.24%	92.74%	99.22%	97.09%	647	141
3	94.42%	96.32%	82.06%	95.75%	97.41%	446	1238
5	89.77%	89.20%	97.95%	97.02%	94.26%	683	246
6	94.91%	92.76%	95.70%	98.20%	98.91%	442	580

Table S8. Performance of Pre-trained CNN with Fine-tuning

Model	Hyper-parameter	Candidate Values
Logistic Regression Models	λ	0.001, 0.01, 0.1, 1, 10, 100, 1000, 10000
One-layer Fully Connected Neural Network	Learning Rate	0.1, 0.01, 0.001, 0.0001, 0.00001
	Batch Size	8, 16, 32, 64
	Neuron Number	16, 64, 128, 512, 1024
LeNet CNN	Learning Rate	0.1, 0.01, 0.001, 0.0001, 0.00001
	Batch Size	8, 16, 32, 64
Pre-trained CNN Off-the-shelf Model	Learning Rate	0.01, 0.001, 0.0001, 0.00001
	Batch Size	8, 16, 32, 64
Pre-trained CNN with Fine-tuning	Learning Rate	0.01, 0.001, 0.0001, 0.00001
	Batch Size	8, 16, 32, 64
	n	1, 2, 3, 4, 5, 6, 7, 8, 9, 10, 11

Table S9. Hyper-parameter Candidates for Grid Search

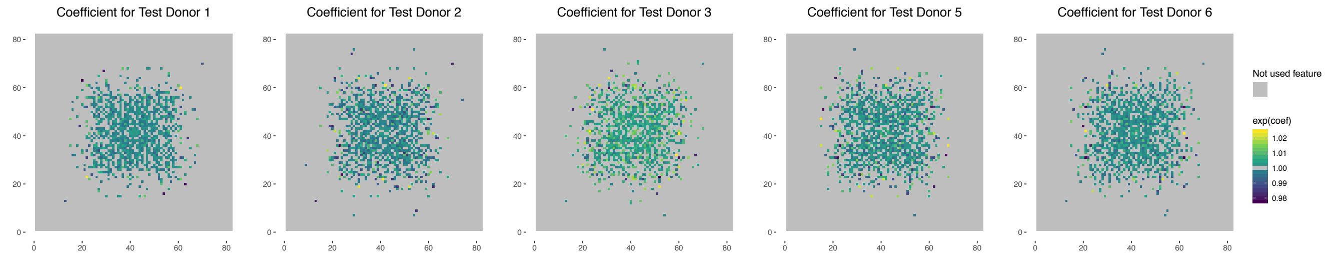

Figure S1. Visualization of odds ratios for one unit increase of each pixel's intensity value. A pixel with odds ratio higher than 1 (light green to yellow) means that, fixing all other pixels, a one-unit intensity increase in this pixel leads to a higher likelihood of predicting the cell is active.

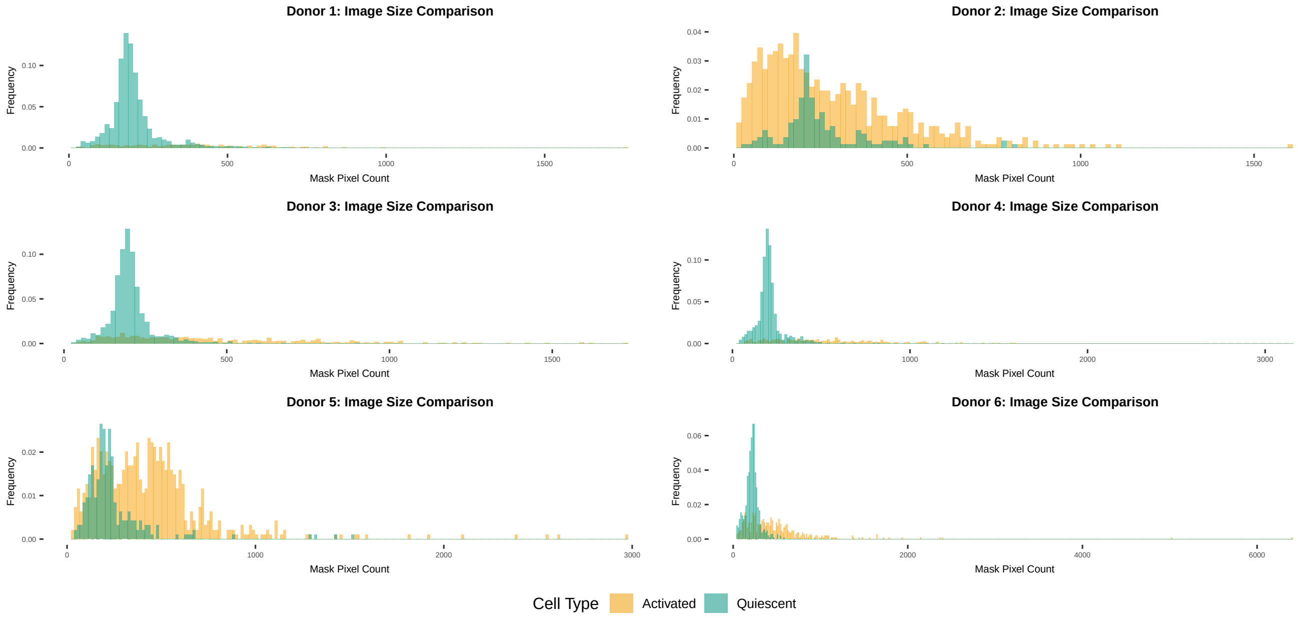

Figure S2. Mask size distribution for each of the donors.

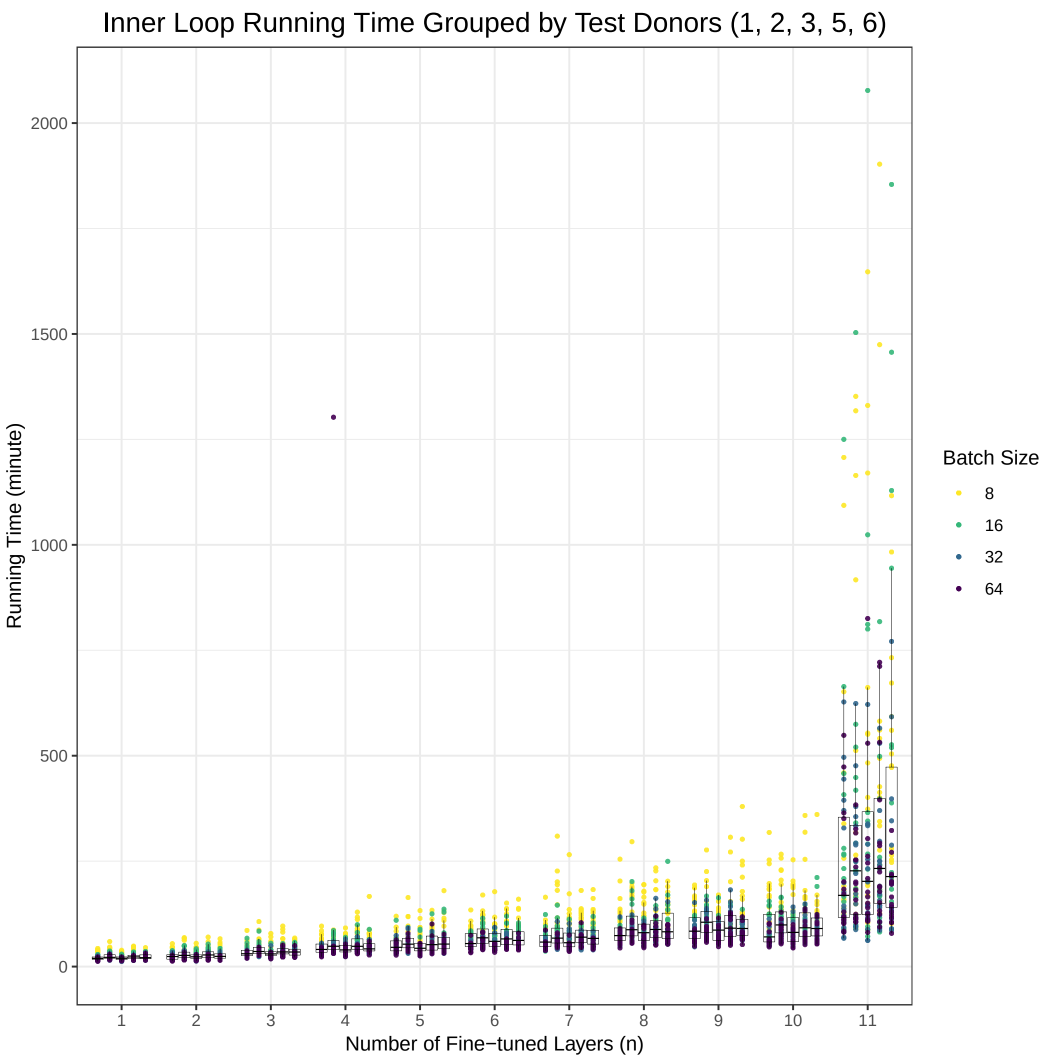

Figure S3. Nested cross-validation inner loop execution time for each n, test donor, and batch size.

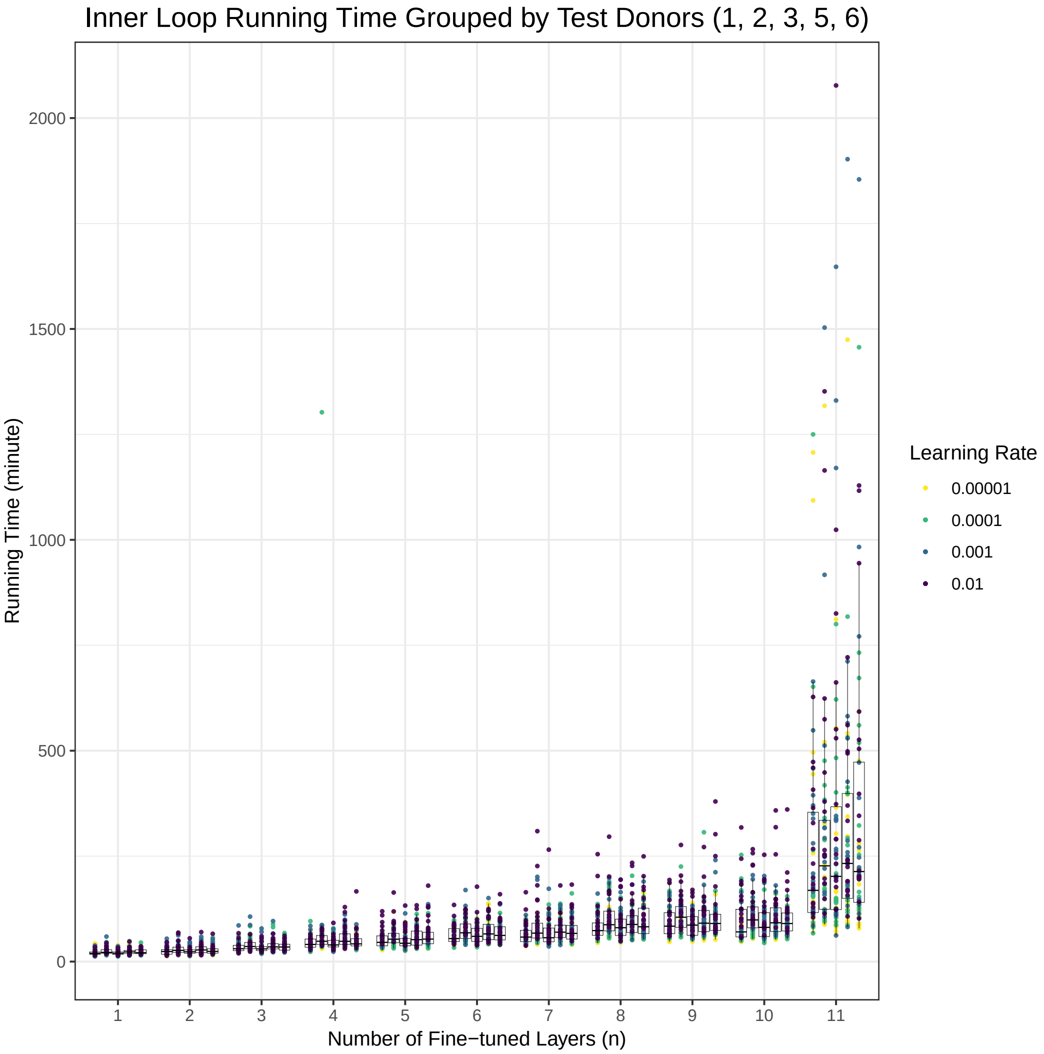

Figure S4. Nested cross-validation inner loop execution time for each n, test donor, and learning rate.

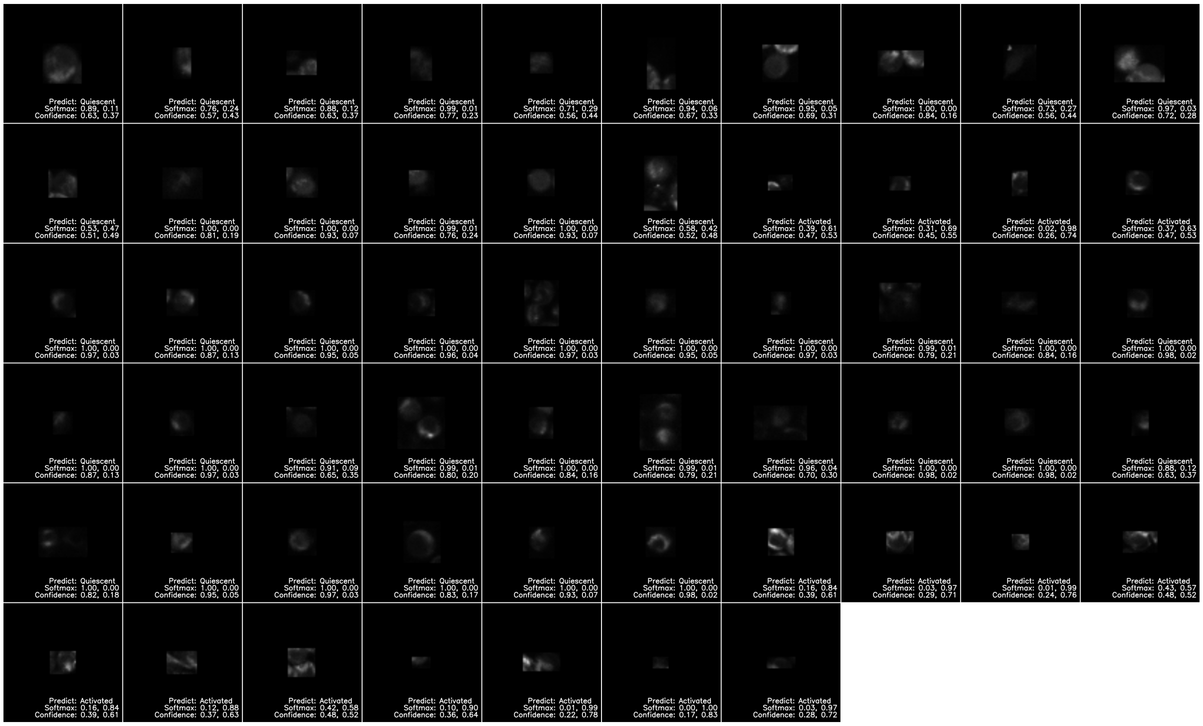

Figure S5. Pre-trained CNN with Fine-tuning Misclassified Images: Donor 1

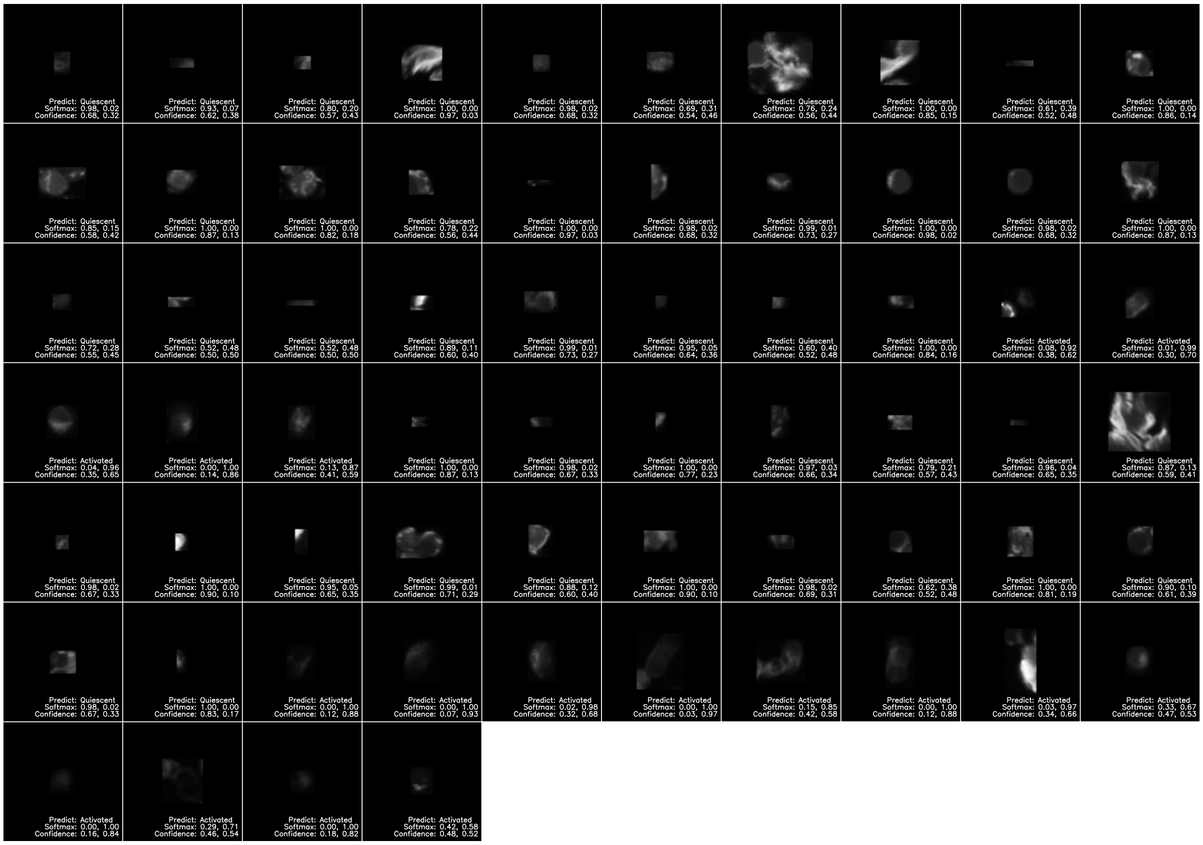

Figure S6. Pre-trained CNN with Fine-tuning Misclassified Images: Donor 2

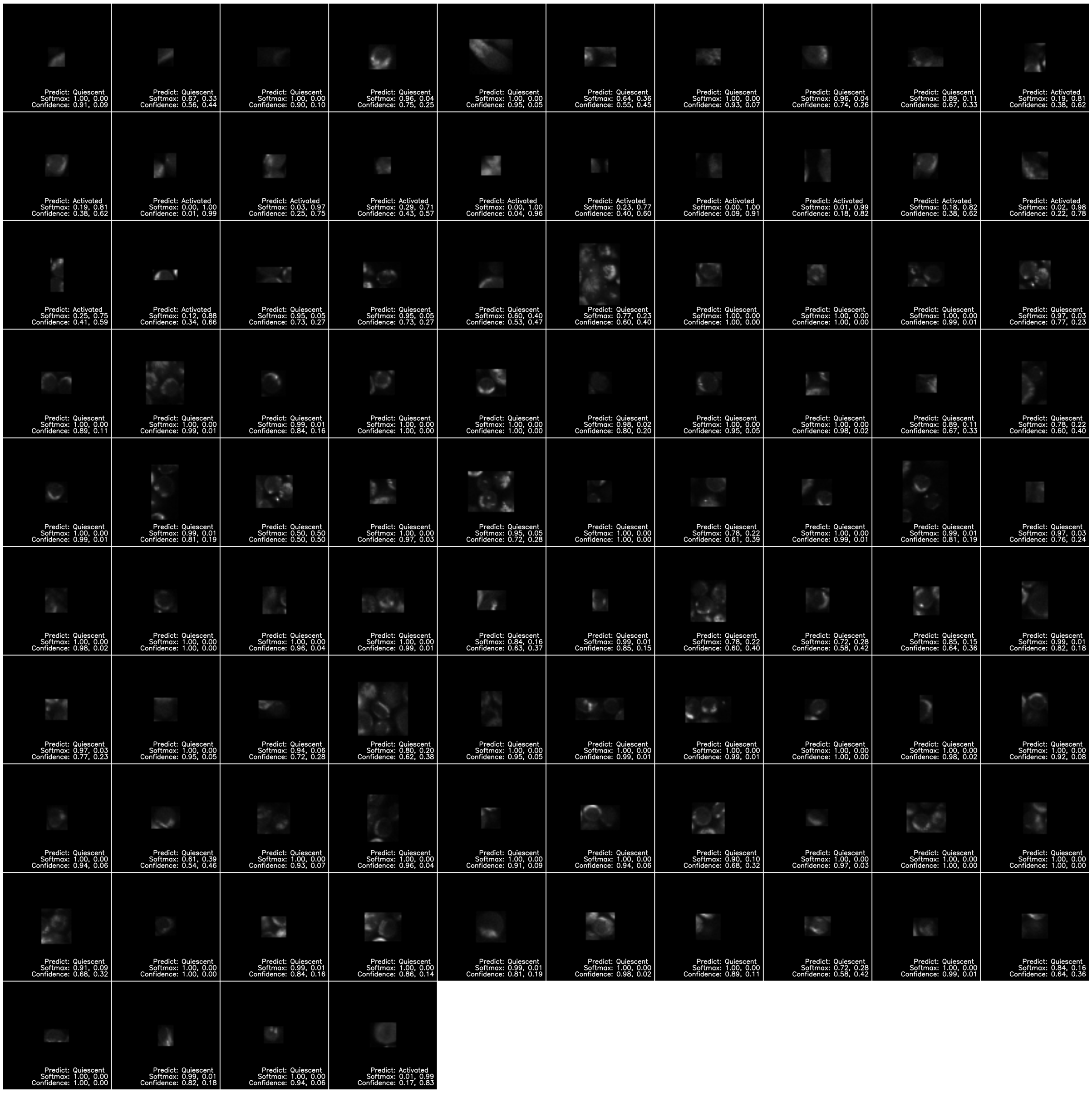

Figure S7. Pre-trained CNN with Fine-tuning Misclassified Images: Donor 3

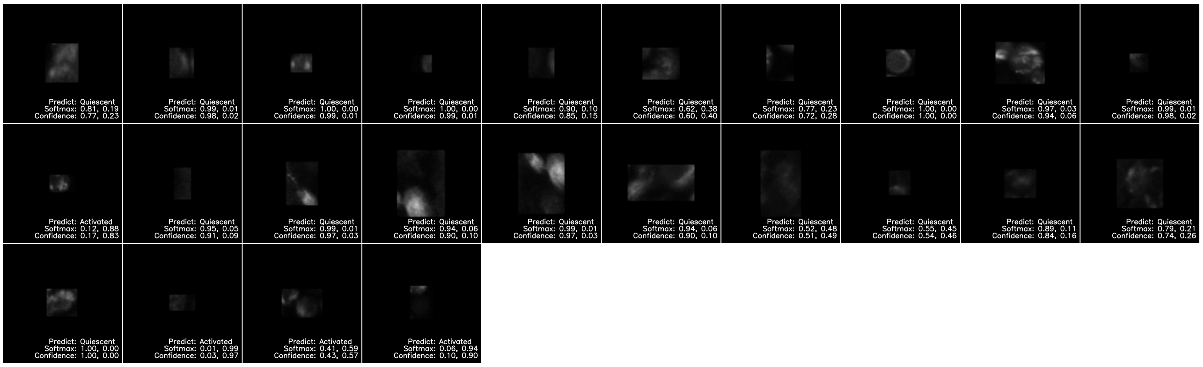

Figure S8. Pre-trained CNN with Fine-tuning Misclassified Images: Donor 4

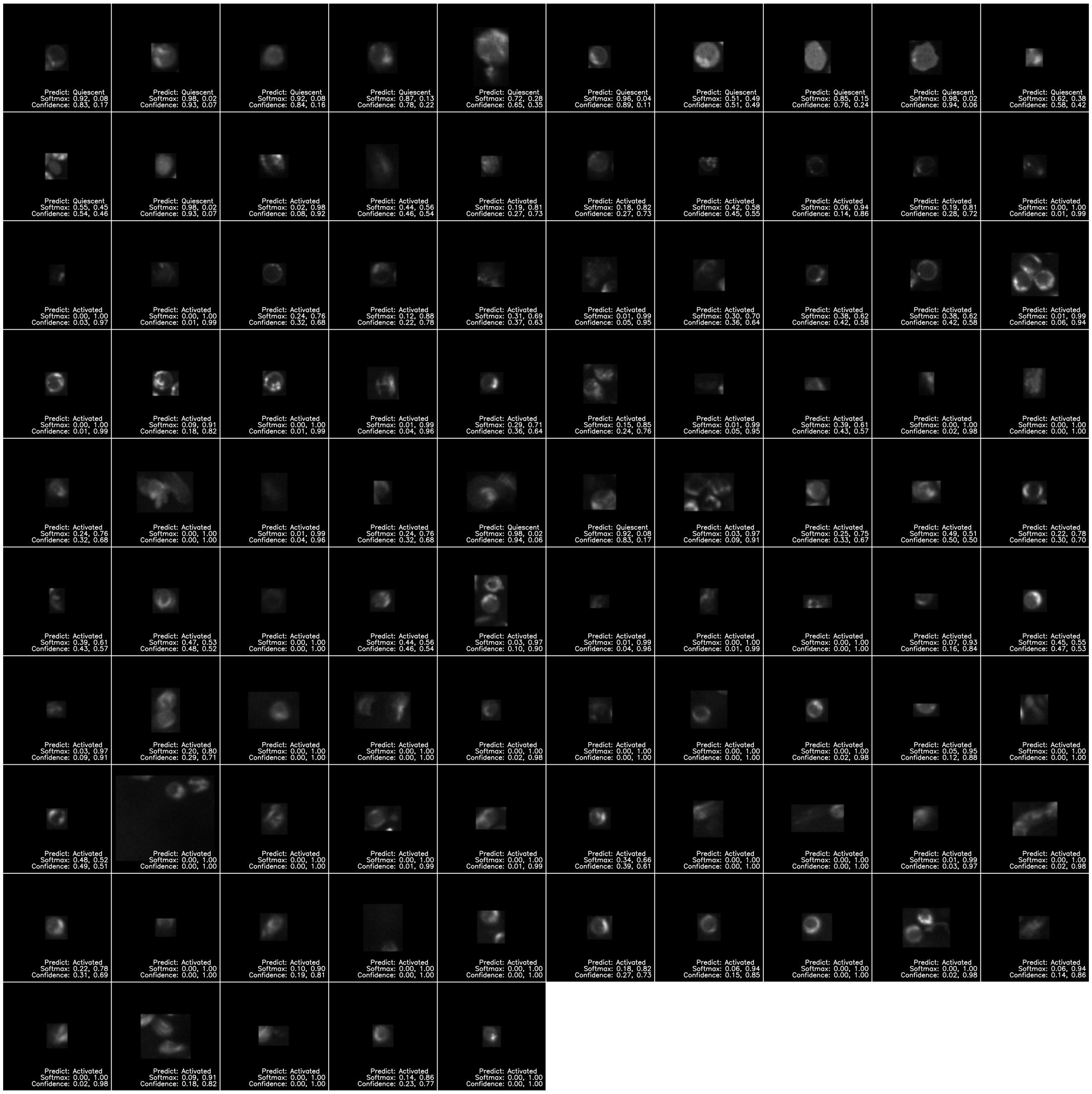

Figure S9. Pre-trained CNN with Fine-tuning Misclassified Images: Donor 5

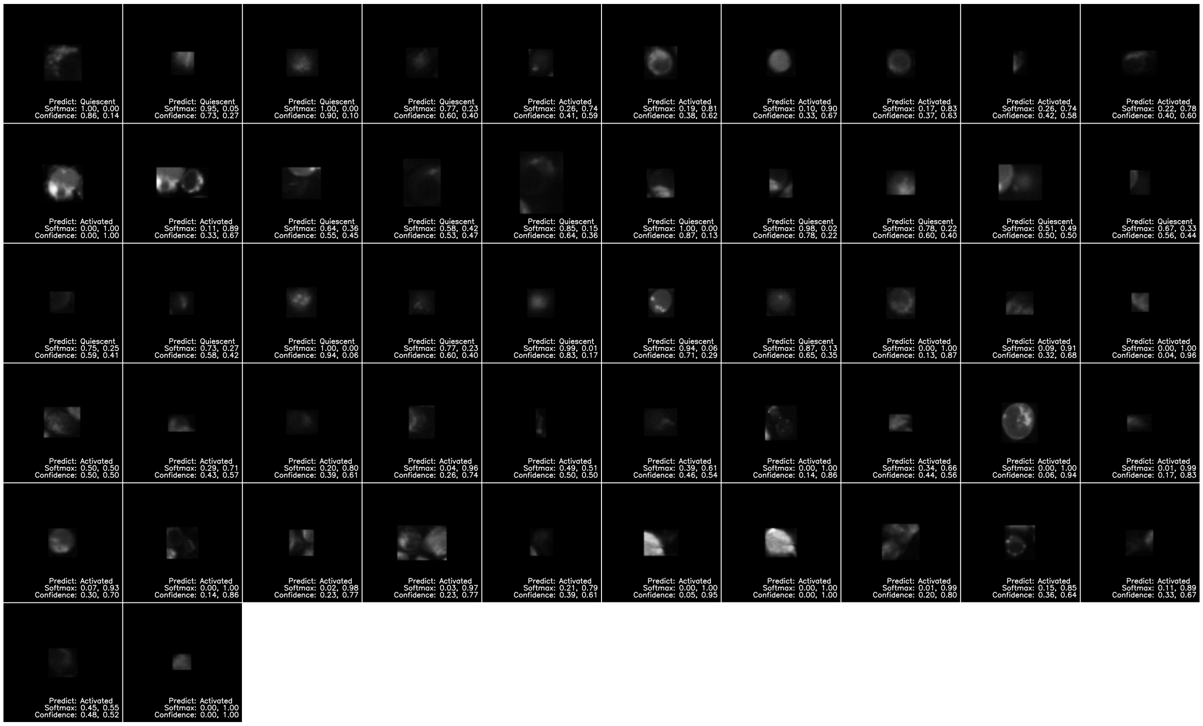

Figure S10. Pre-trained CNN with Fine-tuning Misclassified Images: Donor 6

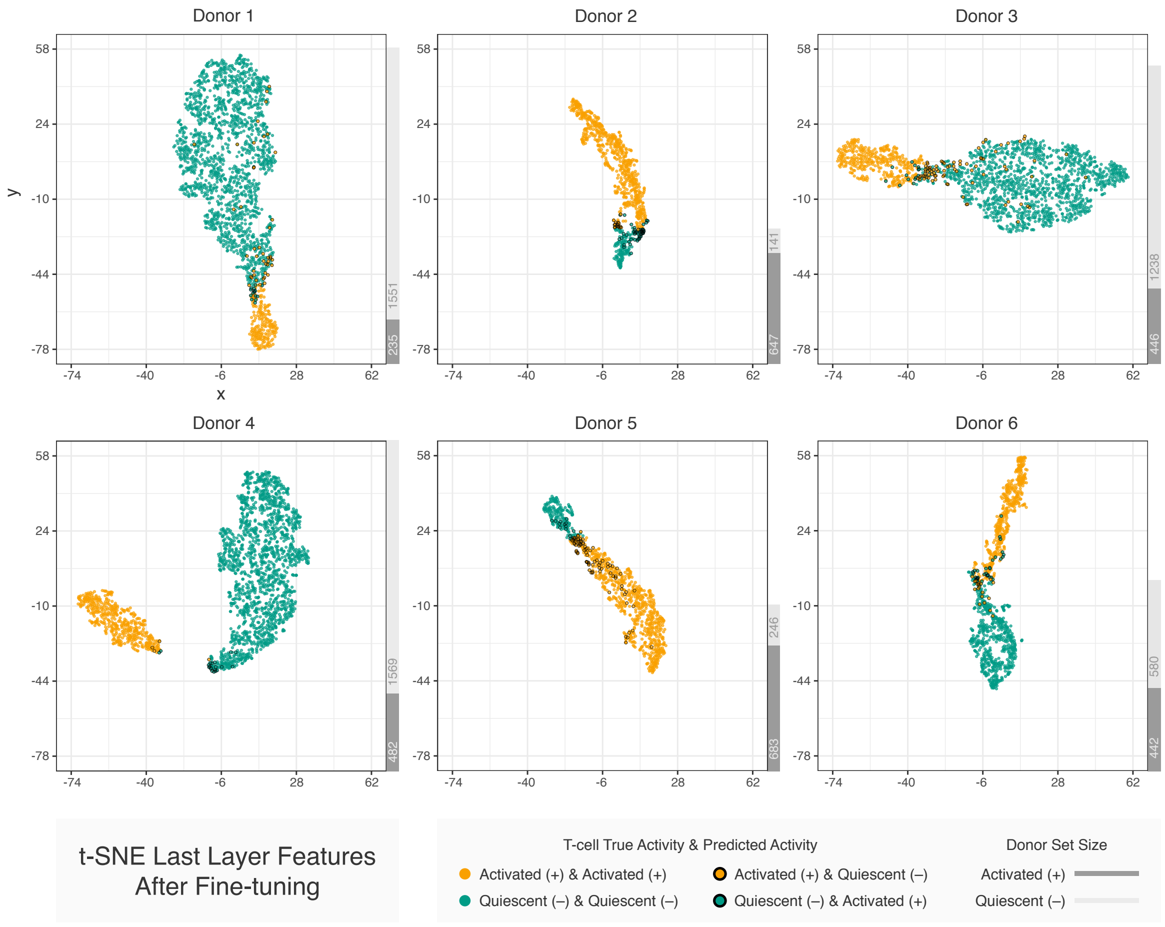

Figure S11. 2D representations of T cell features extracted from the last layer of the pre-trained CNN with fine-tuning. Dimensions were reduced from 2048 using t-SNE. The thick outlines indicate incorrect cell activity state predictions made by the pre-trained CNN with fine-tuning.

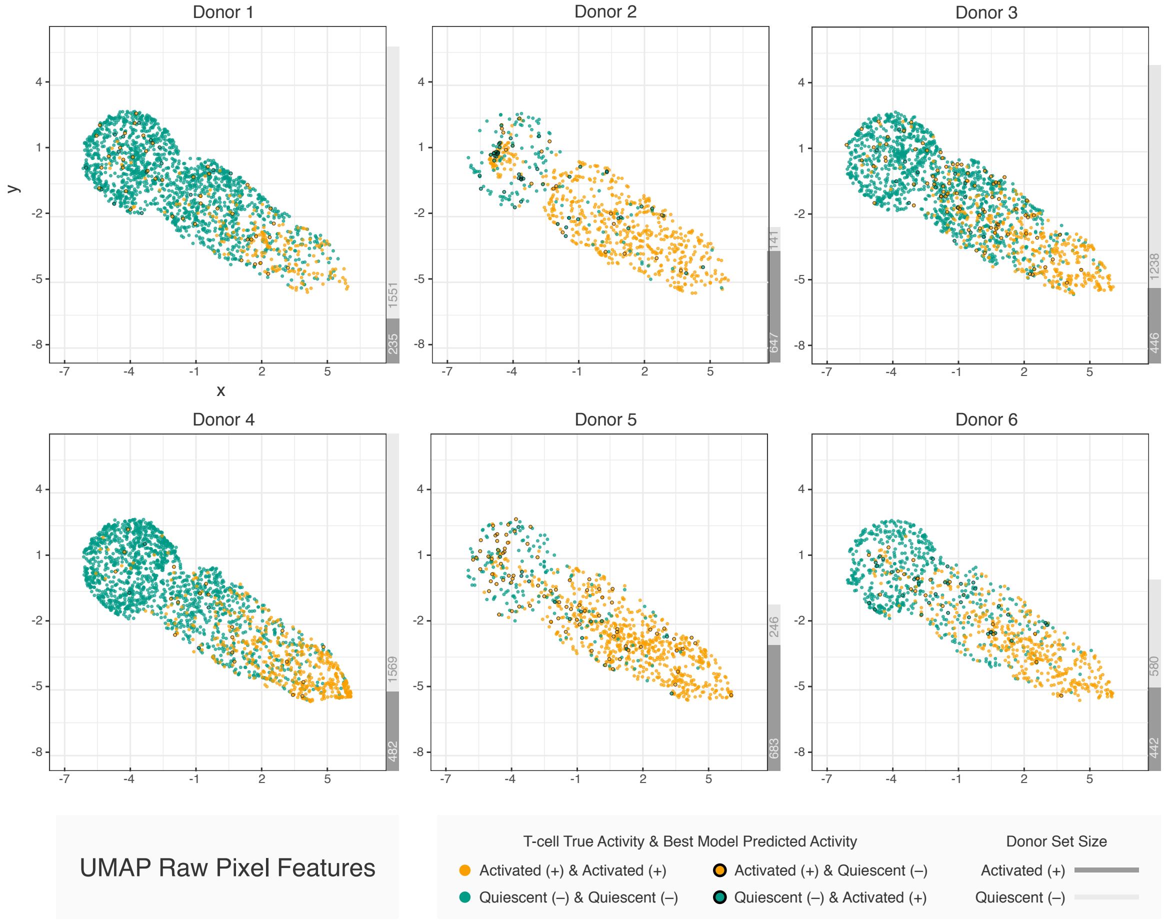

Figure S12. 2D representations of T cell raw pixel features. Dimensions were reduced from $82 \times 82 = 6724$ using UMAP. The thick outlines indicate incorrect cell activity state predictions made by the pre-trained CNN with fine-tuning.

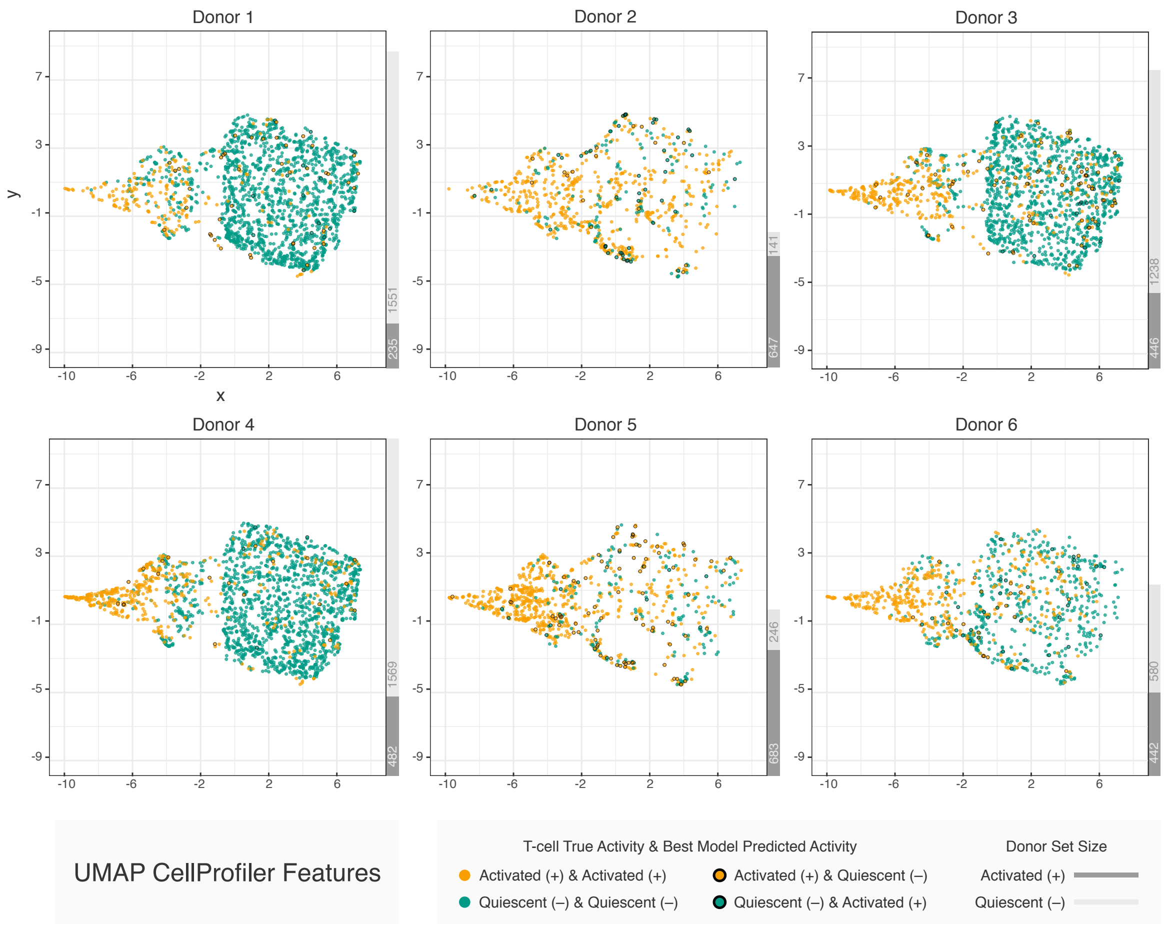

Figure S13. 2D representations of T cell CellProfiler features. Dimensions were reduced from 123 using UMAP. The thick outlines indicate incorrect cell activity state predictions made by the pre-trained CNN with fine-tuning.

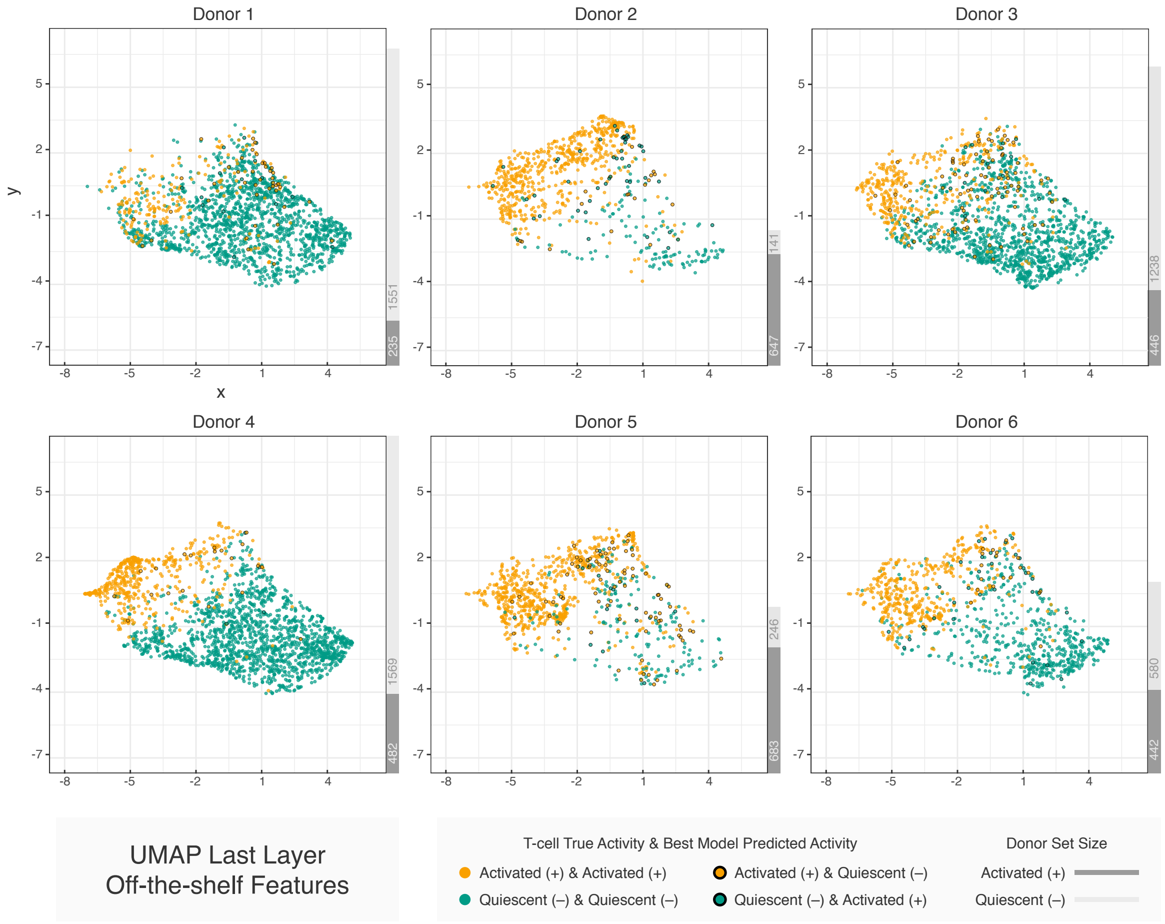

Figure S14. 2D representations of T cell features extracted from the last layer of the pre-trained Inception v3 CNN model before fine-tuning. Dimensions were reduced from 2048 using UMAP. The thick outlines indicate incorrect cell activity state predictions made by the pre-trained CNN with fine-tuning.

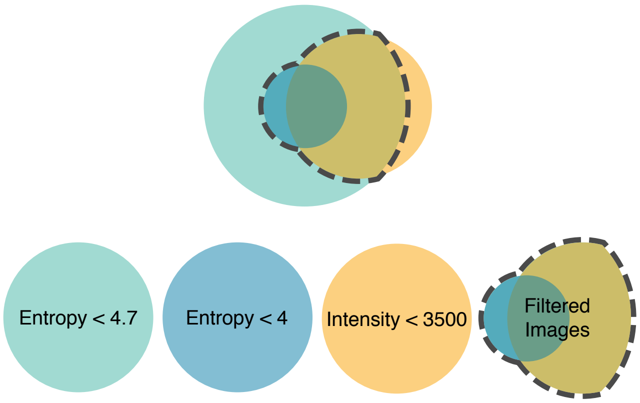

Figure S15. The segmented images are removed from the dataset if their entropy is less than 4 or if their entropy is less than 4.7 and their intensity is less than 3500.

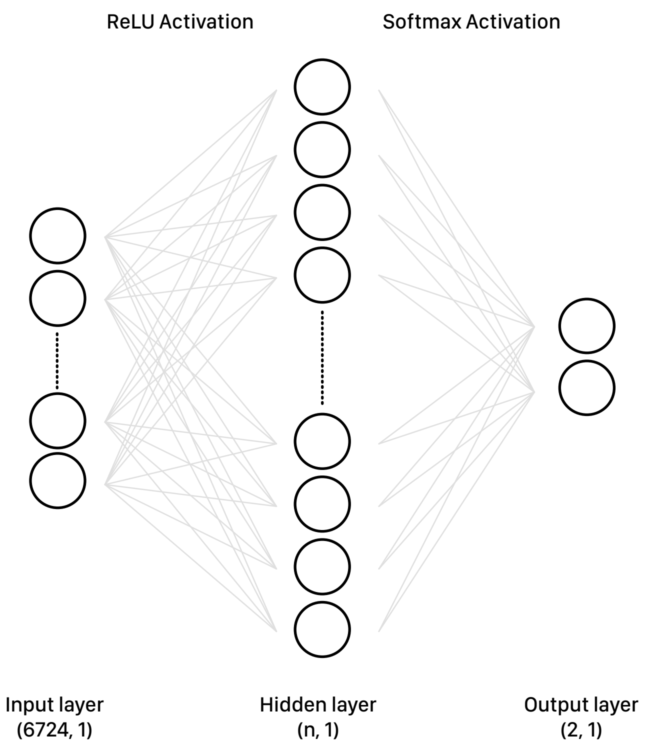

Figure S16. One-layer Fully Connected Neural Network architecture.

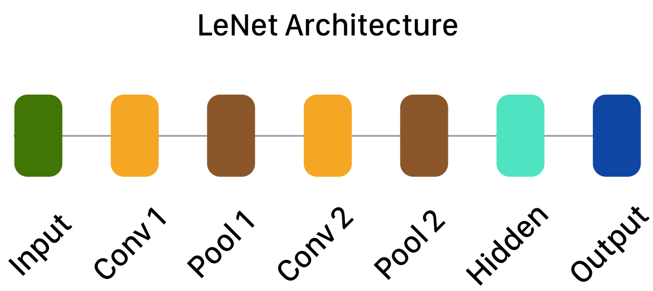

Figure S17. LeNet CNN architecture.
